# Social support from the closest person and sleep quality in later life: Evidence from a British birth cohort study

**DOI:** 10.1016/j.jpsychores.2017.04.014

**Published:** 2017-07

**Authors:** Mai Stafford, Rebecca Bendayan, Ula Tymoszuk, Diana Kuh

**Affiliations:** a MRC Unit for Lifelong Health and Ageing at UCL, London, UK; b Department of Epidemiology and Public Health, UCL, London, UK

**Keywords:** Social support, Pittsburgh Sleep Quality Index, Longitudinal, Ageing, Marital status

## Abstract

**Objectives:**

Supportive social relationships have been found to be related to fewer sleep problems and better sleep quality. We examined associations between positive and negative support from the nominated close person across 15 years of follow-up with sleep quality in older age.

**Methods:**

MRC National Survey of Health and Development study members reported sleep quality at age 68 (n = 2446). Cumulative exposure to and changes in positive and negative support were derived from data at age 53, 60–64 and 68 years. Pittsburgh Sleep Quality Index scores were regressed on social support measures adjusted for i) gender only then additionally ii) education, marital status, number in household, limiting illness, body mass index, caregiving, iii) and affective symptoms.

**Results:**

Greater exposure to positive support and lower exposure to negative support over 15 years were independently associated with better sleep quality at age 68. Sleep quality was poorer for those who experienced declining positive support or increasing negative support. Those who nominated their spouse/partner as their closest person at age 53 but not at age 68 had poorer sleep quality than those who nominated their spouse on both occasions. These associations were not explained by the covariates, including affective symptoms.

**Conclusions:**

Based on repeat data on support from the closest person, this study finds a link between declining social relationship quality and poor sleep quality. Whilst acknowledging that the association may be bi-directional, the study suggests that interventions to improve older people's social relationships may have benefits for sleep.

## Highlights

• We prospectively examined close social relationships and older people's sleep quality.
• Greater cumulative positive support over 15 years was associated with better sleep.
• Independently of this, lower exposure to negative support was linked to better sleep.
• Nominating spouse as the closest person at baseline but not follow-up was linked to poorer sleep.
• Associations were not fully attenuated by education, marital status, illness or depression.

## 1 Introduction

Sleep disorders are more common among older compared with younger adults [1], [2] and, in later life, have been linked to mortality and cardiovascular disease risk [3], [4], physical symptoms, limitations and falls [5], [6], cognitive decline [7] and poor health-related quality of life [8]. Sleep is “embedded within the social world” [9] and supportive social relationships have been found to be related to fewer sleep problems and better sleep quality among middle-aged and older adults [10], [11], [12] as well as in general population samples [13] and working age and occupational samples [14]. However, one national study of older married women found that overall social support across multiple sources was not associated with sleep disturbance, though quality of the marital relationship was [15]. This could indicate that close relationships are particularly relevant for sleep in older age, as others have found [11], possibly because people tend to prioritise meaningful relationships as they age [16] and because of an increased risk of interpersonal life events such as widowhood and caregiving.

Close relationships can entail positive and negative elements. They can be a source of emotional, instrumental and other forms of support but also entail strain and conflict and both positive and negative support have been found to be associated with sleep quality. Negative support is positively correlated with sleep problems [11], [12], [17] though one study found that positive but not negative aspects of the marital relationship were associated with reported sleep quality [18]. One study examined positive and negative aspects of family relationships, finding in an all-age representative sample that both support and strain were related to sleep disturbance when considered singly and that a combination of strain and low support was associated with poorest sleep quality [17]. In an integrative model that considered combinations of positive and negative support, having a higher number of ties characterised by high positivity and low negativity was associated with better sleep quality whilst a higher number of ties characterised by high negativity and low positivity was associated with poorer sleep quality [11].

Studies of social support and sleep in older age tend to rely on cross-sectional designs and do not imply a causal association, nevertheless there are a number of plausible explanatory pathways linking positive and negative support from close persons to sleep in later life. Confiding support improves emotion regulation and facilitates cognitive reappraisal of stressors, linked to reduced likelihood of poor sleep [19]. Other positive aspects of social support, including sense of belonging, shared interests and feeling valued, can all enhance positive mood, which is linked to better sleep [12], [20], [21]. Negative aspects of social relationships, including worries and problems relating to a close person and interpersonal conflict, may lead to ruminating, negative affect and physiological arousal, all linked to poorer sleep [21], [22], [23], [24]. It is of interest to consider the role of affective symptoms since these are intimately linked with sleep. Disturbed sleep is one symptom used to diagnose affective disorder so there is definitional overlap but longitudinal data also indicate a bi-directional link between the two [25].

The aim of the current study is to describe cross-sectional associations between positive and negative social support and sleep quality at age 68 in a large British birth cohort study and also to examine whether changes in social support over the previous fifteen years of follow-up are related to sleep quality. We consider both overall sleep quality and sleep subscales, since associations may differ according to the sleep component of interest [26]. We focus on support derived from the closest person. Gender is a key determinant of sleep [27] and differentiates levels of social support so we test whether gender modifies associations between sleep and support. We also test whether identity of the closest person modifies these associations. Finally, we test whether any associations operate independently of or are explained by depressive symptoms. We hypothesised that positive support would be cross-sectionally associated with higher sleep quality and negative support with poorer sleep quality. We further hypothesised that declines in positive support, increases in negative support and changes in the identity of the closest person would be associated with poor sleep quality.

## 2 Methods

### 2.1 Participants

The Medical Research Council National Survey of Health and Development (NSHD) is a socially stratified sample of all births that occurred during one week in March 1946 across England, Scotland, and Wales. This cohort, based on 5362 births, has been followed prospectively 24 times across life from birth onwards with the latest follow-up in 2014–15 when study members were asked to complete a postal questionnaire at age 68 and then invited to have a home visit by a research nurse at age 69. Of the 2816 people in the target sample living in England, Scotland and Wales, 2370 (84%) completed a postal questionnaire [28]. Of the 126 study members living abroad who remain in contact with the study 83 (66%) returned a questionnaire. No attempt was made to contact the remaining 2420 study members: 957 (18%) had already died, 620 (12%) had previously withdrawn from the study, 448 (8%) had emigrated and were no longer in contact with the study, and 395 (7%) had been untraceable for more than five years. At age 69, study members found to be still living in Great Britain at the last known address or traced to a new address (n = 2698) were invited to have a home visit by a research nurse: 2149 (80%) completed a visit and a further 55 (2%) completed a brief postal questionnaire instead. In total, 2638 study members (94%) provided information on the postal questionnaire and/or completed a home visit. Data for the current study were collected primarily between the ages of 53 and 68–69 years.

### 2.2 Sleep quality

The Pittsburgh Sleep Quality Index has been validated for use in older general population samples [29]. It was included in the postal questionnaire at age 68, captured sleep quality during the previous month and was scored on a continuum from 0 to 21 with higher scores indicating poorer sleep quality. The scale also provides the option of categorising people as having poor or good sleep on the basis of their global score (cut-off <=5/>5) and deriving seven component scores for subjective sleep quality, sleep latency, sleep duration, habitual sleep efficiency, sleep disturbances, use of sleeping medications, and daytime dysfunction (each scored from 0 to 3).

### 2.3 Positive and negative social support

An adapted version of the Close Person's Questionnaire [30] was included at ages 53, 60–64 (self-completed as part of a face-to-face interview) and 68 (by postal questionnaire). Study members were asked to nominate the person they had felt closest to in the last 12 months and respond to six follow-up questions about the quality of that relationship. In confirmatory factor analysis (Appendix A), we found that two scales, one capturing positive support and another capturing negative support, provided a good fit to the data at each age. In order to ensure an equal and comparable definition of both constructs (positive and negative support) over time, longitudinal measurement invariance was examined. The assumption of measurement invariance over time was met as the factor loadings of each indicator held fixed across age, that is, weak measurement invariance TLI > 0.90 and RMSEA < 0.05; Appendix B). Based on this, we derived a positive support score (possible range from 0 to 9) at each age by summing the three items with equal weights and we derived a negative support score in the same way.

### 2.4 Covariates

We controlled for covariates known to be related to sleep, namely education [31], marital status [32], household composition, caregiving [33], physical activity [34], obesity [34] and ill health [35]. All covariate information was provided by the study member at age 68, unless specified. Marital status was grouped as married/cohabiting or unmarried and number of people in household was grouped as one, two, or three plus in the household. Educational attainment was based on formal qualifications reported at age 26 and grouped into none, up to O-level or equivalent (typically gained at age 16), and A-level or higher education. Leisure time activity was grouped as inactive, less active (1–4 occasions taken part in sport or exercise in last month), and more active (5 or more occasions in last month). Limiting long-term illness was indicated if the study member reported an illness or health problem which had lasted for at least 6 months and had limited or severely limited usual activities. Information on the following three covariates was collected as part of a nurse visit at age 69. Body mass index was derived from weight and height measured by trained nurses. Study members also reported if they were providing care to a sick or frail person living in the same household. Affective symptoms were captured by the 28-item version of the General Health Questionnaire and those scoring 5 or more out of a total of 28 were classed as “cases” indicating probable presence of affective disorder.

### 2.5 Statistical analysis

The cross-sectional associations between total PSQI score and positive and negative support were modelled using linear regression in sequential models with adjustment for i) gender only, ii) mutually adjusted plus education, marital status, number of people in household, limiting illness, body mass index and caregiving, and additionally iii) affective symptoms. Since the total PSQI score is right-skewed, we repeated the models using a logarithm transformation of the global sleep quality score and found no material difference in the direction and strength of the associations, hence we present untransformed data for ease of interpretation. We tested for effect modification by gender and by identity of the close person. We additionally present estimates of the main effect of the identity of the close person on total PSQI score. The seven components of sleep were modelled using ordinal logistic regression.

We included longitudinal data on social support in two ways. First, we derived cumulative exposure to high positive and high negative support (based on number of occasions the study member reported support in the top third, ranging from 0 to 3). We also examined within-person trajectories of positive and negative support in a two stage model. First, we estimated intercept and change in positive support between age 53 and 68–69 years using a random slopes model with an unstructured variance-covariance matrix for the random parameters. Initially we included linear age and gender as fixed and random parameters to be estimated but gender did not explain between-person variation in support and was dropped. Person-level random intercept (centred at age 53) and slope estimates (indicating person-specific change in positive support) were generated. In a similar way, we estimated intercept and change in negative support between age 53 and 68–69 years. These person-specific intercepts and slopes were then standardized and used as independent variables in a linear regression model with adjustment for the covariates. In addition, we examined associations between total PSQI score and change in identity of the close person from age 53 to age 68.

A total of 2446 study members provided data on at least one sleep subscale plus concurrent social support and 2100 of these provided complete sleep data (to allow calculation of the total PSQI score). Of these, 1951 provided longitudinal social support data. Covariate data were rarely missing if collected by postal questionnaire (< 2% for most variables, 3% for education) but more commonly missing if collected at the nurse visit (18% for caregiving, 19% for affective disorder and 20% for obesity). Using full information maximum likelihood estimation under the assumption that data were missing at random, those with missing covariate data were included in the analysis. Auxilliary variables (obesity, caregiving and affective disorder at the previous data collection) were included since this can reduce bias and increase efficiency. Compared with all those who participated at age 68–69, those with valid sleep and social support data were more commonly married (p = 0.08), in a two-person household, with higher educational attainment, without long-term health limitations, physically active, and without affective disorder, but they did not differ in obesity prevalence or caregiving. Analyses based on complete cases did not materially differ from those presented here (though standard errors were somewhat larger in magnitude). Compared with those included in only the cross-sectional analysis, those who provided longitudinal social support data were more commonly married, with higher educational attainment, without health limitation, and without affective disorder. Stata (SE version 14) and Mplus (version 6) were used for the linear and ordered logistic regression analyses respectively.

## 3 Results

On average, women had poorer sleep quality than men (Table 1). The majority of study members nominated their spouse or partner as their closest person, with this percentage being higher for men (85.4%) than women (68.8%, p < 0.001). Over 40% of study members had a limiting long-term illness, though this was severely limiting for only 5.3%. The overall prevalence of affective disorder was 12.7% and was higher for women than men.

**Table 1:** Characteristics of the study sample at age 68 based on 2100 study members with complete sleep data.

	All (n = 2100)	Men (n = 1036)	Women (n = 1064)	P for sex difference
	Mean (sd) Median (min, max)	Mean (sd) Median (min, max)	Mean (sd) Median (min, max)	
PSQI global score	5.0 (3.2)	4.4 (2.9)	5.6 (3.4)	⁎⁎
	4 (0,17)	4 (0,17)	5 (0,17)	
	%	%	%	
Identity of closest person				⁎⁎
Spouse/partner	77.0	85.4	68.8	
Son/daughter	11.1	6.2	15.8	
Other relative	3.5	2.4	4.6	
Friend or neighbour	5.1	3.0	7.1	
Other	2.5	2.1	2.9	
No-one close	0.8	0.9	0.8	
Married or cohabiting	76.6	81.8	71.6	⁎⁎
Single, divorced or widowed	23.4	18.2	28.4	
Living alone	17.5	13.9	21.0	⁎⁎
Two person household	73.5	75.4	71.6	
Three or more in household	9.1	10.8	7.4	
Low educational attainment	31.5	32.1	30.8	⁎⁎
Medium educational attainment	28.7	21.0	36.3	
High educational attainment	39.8	46.9	32.9	
No limiting long-term illness	58.8	60.3	57.4	
Limited but not severely	35.9	34.8	37.0	
Severely limited	5.3	5.0	5.7	
Physically inactive	59.1	58.6	59.5	
Less active (1–4 occasions per month)	13.0	11.9	14.1	
More active (5 + occasions per month)	27.9	29.5	26.4	
Body mass index; mean (SD)	28.0 (5.1)	28.0 (4.5)	28.0 (5.6)	
Caregiving within the household				
Yes	9.6	8.6	10.5	
No	90.4	91.4	89.5	
Affective disorder				⁎⁎
Yes	12.7	8.7	16.7	
No	87.3	91.3	83.3	

⁎⁎ p < 0.001.

In a gender-only adjusted model, each 1 standard deviation increase in positive support from the closest person was associated with a difference of − 0.55 (95% CI − 0.41, − 0.68) units on the global sleep quality score indicating better sleep quality among those with more positive support (Table 2 model 0). The corresponding estimate for negative support was 0.51 (95% CI 0.37, 0.64). With both social support scales included together and adjusted for covariates, an inverse association between positive support and global sleep quality remained with some attenuation (model 1). Someone with a positive support score 2 standard deviations above the mean would be expected to have a mean sleep quality score 1.48 points lower than someone with a positive support score 2 standard deviations below the mean, controlling for demographic, lifestyle and health-related variables. More negative support from the closest person was associated with poorer sleep. These associations remained on further adjustment for affective disorder (model 2). There was no evidence that the association between support and sleep was modified by gender or by identity of the close person (data available from the authors).

**Table 2:** Cross-sectional associations between sleep quality and support from the closest person at age 68.

	PSQI score model 0^a^	PSQI score model 1^b^	PSQI score model 2^c^	Shorter sleep duration^c^	Greater sleep disturbance^c^	Longer sleep latency^c^	Greater daytime sleepiness^c^	Poorer sleep efficiency^c^	Perceived sleep quality^c^	Needs medication to sleep^c^
	n = 2100	n = 2100	n = 2100	n = 2398	n = 2224	n = 2266	n = 2420	n = 2384	n = 2433	n = 2422
	Coeff (se)	Coeff (se)	Coeff (se)	OR (95% CI)	OR (95% CI)	OR (95% CI)	OR (95% CI)	OR (95% CI)	OR (95% CI)	OR (95% CI)
Positive support (per 1 sd increase)	− 0.55 (0.07)⁎⁎	− 0.37 (0.07)⁎⁎	− 0.36 (0.07)⁎⁎	0.91 (0.86, 0.96)⁎⁎	0.99 (0.94, 1.04)	0.93 (0.88, 0.97)⁎	0.89 (0.84, 0.96)⁎⁎	0.92 (0.88, 0.97)⁎⁎	0.89 (0.85, 0.93)⁎⁎	0.93 (0.87, 1.00)⁎
Negative support (per 1 sd increase)	0.51 (0.07)⁎⁎	0.36 (0.07)⁎⁎	0.32 (0.07)⁎⁎	1.02 (0.97, 1.08)	1.11 (1.05, 1.17)⁎⁎	1.05 (1.00, 1.10)⁎	1.19 (1.14, 1.24)⁎⁎	1.03 (0.98, 1.07)	1.10 (1.05, 1.15)⁎⁎	1.10 (1.03, 1.18)⁎

a Model includes gender and each support scale singly.

b Both positive and negative support included together and adjusted for marital status, number people in household, educational attainment, longstanding limiting illness, leisure time physical activity, body mass index, caregiving.

c Additionally adjusted for affective disorder.

⁎⁎ p < 0.001.

⁎ p < 0.05.

Turning to the individual sleep subscales, positive support showed a protective association with sleep duration, sleep latency, daytime sleepiness, sleep efficiency, perceived sleep quality and sleep medication (Table 2). Negative support was associated with an increased risk of greater sleep disturbance, longer sleep latency, greater daytime sleepiness, poorer perceived sleep quality and needing medication to sleep. For each sleep subscale, one or both social support measures showed a statistically significant association.

**Fig. 1:**
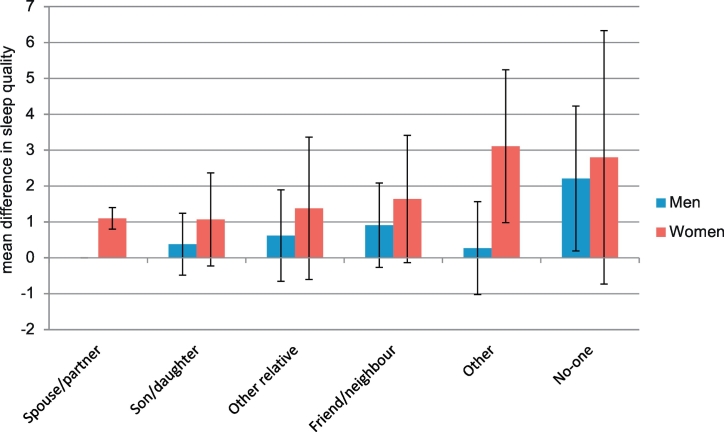
Sleep quality by identity of close person at age 68.

Compared with the reference group of those who nominated their spouse/partner as the close person, those who nominated another source had poorer sleep quality though this attained statistical significance in only two groups and was modified by gender (Fig. 1, Appendix C). Men who did not have a close person had a mean score of 2.20 (95% CI 0.20, 4.20) points higher than those who nominated their spouse/partner. This association was not statistically significantly different in women. Women who did not have a close person also had poorer sleep quality though the confidence interval indicates slightly greater imprecision among women. In addition, women who nominated someone other than a family member or a friend/neighbour had poorer sleep quality (indicated by a higher PSQI score) than those who nominated their spouse/partner. This association was not seen in men (p for gender interaction = 0.03). These associations remained on additional adjustment for affective disorder (with only slight attenuation; Appendix C model 2).

Positive support from the closest person was similar at ages 53 and 68 for men but was a little lower at age 68 for women (Table 3). For both men and women, positive support was highest at age 60–64. There was a graded association between number of occasions positive support was in the top third and higher sleep quality (Table 4). The association between number of occasions with high negative support and sleep was not strictly graded but there was clear evidence that those exposed to negative support on more occasions had poorer sleep than those who were less exposed. Inclusion of both social support scales together and adjustment for covariates did not fully attenuate these associations.

**Table 3:** Positive and negative support at ages 53, 60–64 and 68 years for study members with complete sleep data at age 68.

	Age 53	Age 60–64	Age 68
Men	(n = 899)	(n = 801)	(n = 1036)
Positive support; mean (sd)	6.55 (1.82)	6.78 (1.76)	6.52 (1.95)
Median (min, max)	7 (0,9)	7 (1,9)	7 (0,9)
Negative support; mean (sd)	1.89 (1.62)	1.79 (1.46)	1.43 (1.37)
Median (min, max)	2 (0,9)	2 (0,8)	1 (0,9)
Identity of closest person (%)			
Spouse/partner	89.2	92.5	85.4
Son/daughter	2.7	4.1	6.2
Other relative	2.9	3.0	2.4
Friend or neighbour	2.7	0.0	3.0
Other	1.9	0.3	2.1
No-one close	0.7	0.1	0.9
Women	(n = 956)	(n = 830)	(n = 1064)
Positive support; mean (sd)	6.42 (1.80)	6.59 (1.74)	6.22 (1.91)
Median (min, max)	6 (0,9)	7 (1,9)	6 (1,9)
Negative support; mean (sd)	1.73 (1.62)	1.93 (1.61)	1.71 (1.58)
Median (min, max)	1 (0,9)	2 (0,9)	1 (0,9)
Identity of closest person (%)			
Spouse/partner	79.8	81.3	68.8
Son/daughter	9.4	14.5	15.8
Other relative	4.4	3.9	4.6
Friend or neighbour	4.3	0.0	7.1
Other	1.9	0.1	2.9
No-one close	0.2	0.2	0.8

**Table 4:** Sleep quality at age 68 and longitudinal data on support from the closest person at ages 53, 60–64 and 68.

	PSQI score model 0^a^	PSQI score model 1^b^	PSQI score model 2^c^
	n = 1951	n = 1951	n = 1951
	Coeff (se)	Coeff (se)	Coeff (se)
*Positive support: occasions in top third*			
0	Reference	Reference	Reference
1	− 0.34 (0.22)	− 0.19 (0.22)	− 0.18 (0.21)
2	− 0.83 (0.23)	− 0.51 (0.23)⁎	− 0.45 (0.22)⁎
3	− 1.25 (0.22)	− 0.89 (0.22)⁎⁎	− 0.84 (0.22)⁎⁎

*Negative support: occasions in top third*			
0	Reference	Reference	Reference
1	0.97 (0.22)⁎⁎	0.79 (0.22)⁎⁎	0.76 (0.22)⁎⁎
2	0.87 (0.23)⁎⁎	0.69 (0.23)⁎⁎	0.63 (0.23)⁎
3	1.74 (0.23)⁎⁎	1.32 (0.24)⁎⁎	1.17 (0.24)⁎⁎

*Positive support trajectories*			
Intercept (age 53)	− 0.51 (0.07)⁎⁎	− 0.35 (0.07)⁎⁎	− 0.34 (0.07)⁎⁎
Slope (change age 53–68)	− 0.25 (0.06)⁎⁎	− 0.16 (0.06)⁎	− 0.16 (0.06)⁎

*Negative support trajectories*			
Intercept (age 53)	0.64 (0.08)⁎⁎	0.41 (0.08)⁎⁎	0.36 (0.08)⁎⁎
Slope (change age 53–68)	0.22 (0.08)⁎	0.15 (0.08)⁎	0.14 (0.08)

*Change in identity of closest person*			
Spouse/partner at age 53 and 68	Reference	Reference	Reference
Spouse/partner at age 68 only	0.02 (0.34)	0.34 (0.33)	0.35 (0.33)
Spouse/partner at age 53 only	0.63 (0.23)⁎	0.81 (0.28)⁎	0.72 (0.28)⁎
Spouse/partner not nominated	0.23 (0.24)	0.43 (0.32)	0.30 (0.31)

a Model includes gender and each support scale singly.

b Both positive and negative support included together and adjusted for marital status, number people in household, educational attainment, longstanding limiting illness, leisure time physical activity, body mass index, caregiving.

c Additionally adjusted for affective disorder.

⁎⁎ p < 0.001.

⁎ p < 0.05.

Analysis of within-person trajectories of social support from ages 53 to 68 showed that women had a lower level of positive support at baseline compared with men. On average positive support decreased with age for women but did not change with age not for men (Appendix D). Baseline positive support and age-related change in positive support varied significantly between study members. A random slope model also fitted the data for negative support. This showed that men, but not women, experienced a decline in negative support with age. At the next stage of the longitudinal analysis, with intercept and slope residuals included as independent variables, we found that greater positive support at baseline (age 53) and greater increase in positive support (from age 53 to 68) was associated with better sleep quality at age 68 (Table 4). Similarly, higher baseline negative support and greater increase in negative support ages 53 to 68 were associated with poorer sleep quality. These associations were only partly attenuated on adjustment for covariates.

The percentage nominating a son or daughter as the closest person increased across the ages (Table 3). Altogether, the spouse/partner was nominated at both ages 53 and 68 by 71.5% of study members, only at age 53 by 12.2%, only at age 68 by 5.2%, and 11.0% did not nominate a spouse/partner at either age. Compared with those who nominated the spouse/partner as the closest person at both ages, those who nominated the spouse/partner only at age 53 had poorer sleep quality (Table 4). Controlling for affective disorder explained only a small part of this association. Further investigation revealed that 62% of the group who nominated their spouse/partner only at age 53 were widowed by age 68 in contrast to only 8% of the total sample.

## 4 Discussion

Based on data from a large, representative cohort study set in Britain, we found that positive support from the closest person was related to better sleep quality and negative support to poorer sleep quality in older age, controlling for sociodemographic characteristics and long-term illness. Additional adjustment for affective disorder only slightly attenuated these associations. Using longitudinal data, we found that greater cumulative exposure over 15 years of follow-up to high positive support was associated with better sleep quality and those with greater exposure to negative support had poorer sleep. Considering trajectories of support over 15 years, we found that increasing positive support was associated with better sleep at age 68 and increasing negative support with poorer sleep. Those nominating the spouse/partner as the closest person at age 53 but not at age 68 had significantly poorer sleep quality compared to those nominating the spouse/partner at both ages.

Cross-sectionally, we found that those who nominated a spouse or partner as their closest person tended to have better sleep quality than those who nominated another person, controlling for marital status. This aligns with an all-age study set in Asia which showed that not consulting a spouse or partner in the first instance regarding personal problems and important matters was associated with greater likelihood of sleep problems [36]. We also found that not having felt close to anyone in the last twelve months was related to poorer sleep quality. Though we did not directly measure loneliness, this finding aligns with an earlier study demonstrating an inverse association between loneliness and older people's sleep quality [20].

One previous study have found that positive but not negative marital quality was associated with sleep [18] and another that poorest sleep was seen for those with a combination of low support and high social strain [17]. Integrating positive and negative aspects of relationships into a single indicator, Kent and colleagues [11] showed that having a greater number of predominantly supportive ties was related to better sleep, having more predominantly negative ties was related to poorer sleep, and having more ambivalent ties characterised by high levels of both positivity and negativity was not related to sleep quality. Findings from these different studies cannot be directly compared, however, since they considered different social relationships, namely family outside the household [17], the marital relationship [18] and here the nominated closest person (though this frequently was the spouse or partner). We found that both positive and negative aspects of the close relationship were independently associated with sleep and extend earlier studies by considering cumulative exposure to positive and negative support as well as support trajectories using longitudinal data.

The results from our analysis of repeat support data to capture cumulative exposure suggest that long-term as well as recent social support is related to sleep quality. Chronic exposure to high negative support was most strongly associated with poor sleep. Using repeat data to estimate trajectories of social support suggests that changes in support also matter for sleep quality. Independently of the starting point, those who experienced increasing negative support over time had poorer sleep. Longitudinal data on social support at work (e.g. [14], [37]. and on the quality of the parent-adolescent relationship [38] has previously been assessed in relation to sleep in occupational and adolescent samples respectively but we are not aware of studies utilising longitudinal on social support in an ageing cohort. Using repeat data on the identity of the nominated close person highlighted that “losing” the spouse/partner as the closest person was linked to poorer sleep but those who “gained” a spouse/partner as the closest person did not differ from those who continually nominated their spouse/partner. This may reflect the impact of deterioration in the quality of the spousal relationship on sleep quality [39]. It may also reflect the impact of being widowed [40], [41] and of transition to widowhood. Troxel and colleagues found poorer subjective sleep quality among those who became widowed over eight years of follow-up but no difference in subjective sleep quality between those who gained a partner and those who were continually partnered [42]. The experience of widowhood can affect sleeping patterns directly since co-sleeping is associated with better subjective sleep quality (though not necessarily with objectively assessed sleep [43]). Becoming widowed and deteriorating spousal relationship quality are also associated with increased risk of affective disorder, discussed below. Loss of a partner is also associated with loss of behavioural regulators including diet, appetite and regular sleep time and with heightened activity in the sympathetic-adrenal-medullary and the hypothalamic-pituitary-adrenal axes, each of which may affect sleep quality [44]. A fourth possibility for the association between loss of spouse/partner as the close person and sleep is a shared underlying health risk among spouses that is not adequately captured by limiting long-term illness in our analysis.

Affective disorder is a strong and consistent predictor of poor sleep quality [35] and may mediate the association between poor social support or other indicators of suboptimal social relationships and sleep. Our results revealed an association between social support and sleep that remained on adjustment for symptoms of depression and anxiety, in line with one other study which considered loneliness and sleep [45]. Kent et al. [11] found depression significantly mediated the association between support and sleep though it is unclear whether a direct association between support and sleep also remained. Additional or alternative pathways are plausible. Lower positive and more negative support may affect sleep also through increased rumination, which is moderately positively correlated with symptoms of depression and anxiety but shows an association with sleep quality independently of these affective symptoms [46]. Social support may also influence emotion regulation [47] and emotional reactivity is implicated in the development of sleep problems [48]. Particularly in the context of co-sleeping, emotional (and physical security) is linked to down-regulation of watchfulness, thus facilitating sleep [49]. However, we were unable to test these explanatory pathways in these data or to examine directionality. Poor sleep, possibly through increased daytime fatigue, may lead to impaired functioning including impaired social life and greater difficulty in managing conflict within social relationships [50] and greater emotional reactivity [51]. Sleep and relationship quality are likely to affect each other although not all studies have found differences in social support by presence of insomnia [52] and one longitudinal study found loneliness to predict future sleep quality but not vice versa [26].

Exploratory analysis of the PSQI sleep subscales generally indicated that social support was associated with each component of sleep quality and did not indicate a clear role for a particular kind of social support with any specific sleep subscale. Some other studies have suggested that sleep disturbance or fragmentation but not sleep duration may be particularly closely related to social relationship adversity. One showed that loneliness, which they hypothesised reflected a heightened sense of vulnerability, was related to more fragmented sleep but not shorter sleep duration [53] but this has not been supported elsewhere [45]. Negative but not positive aspects of the marital relationship have been associated with reported trouble sleeping (based on long sleep latency, sleep disturbance and not feeling rested) [32]. In that study, neither positive nor negative aspects were related to reported sleep time.

In interpreting these findings, a number of limitations should be considered. We did not have data on sleeping arrangements though we controlled for marital/cohabitation status and most adults co-sleep with their partner [52]. We used self-reported sleep data, not polysomnography. It has been suggested that the PSQI captures general affective symptoms rather than actual sleep parameters [54], though some studies find a weak to moderate positive correlation between the PSQI and objective sleep measures [55] and it should be noted that our results were not fully explained by adjustment for affective symptoms. One community-based study found positive marital quality was associated with less sleep fragmentation assessed by actigraph but not with reported sleep disturbance [15]. They suggested that relationship quality may influence perceived sleep and actigraph-assessed sleep in different ways and speculated that relationship quality may affect the type of sleep (e.g. deep versus light sleep). On the other hand, reported sleep quality captures the impact of sleep on daily life and several studies indicate consistency in the association with health across both kinds of sleep instruments [56]. We considered support from only the closest person and did not have longitudinal data to explore a wider set of social ties or other aspects of social relationships that have been linked to sleep including satisfaction with social life [57], attachment anxiety and avoidance [58] and loneliness [26], [45], [59].

Research is beginning to identify interventions to reduce loneliness and improve the quality of older people's social relationships. Our findings, though currently based on observational data, suggest that improvements to positive support and reductions in negative support may have the potential to improve sleep quality. The public health benefits may be substantial given the pervasiveness of poor sleep among older people and the impact of poor sleep on quality of life and other health outcomes.

## Competing interests

All authors have completed the Unified Competing Interest form at http://www.icmje.org/coi_disclosure.pdf. The authors have no competing interests to report.

## Figures and Tables

**Table:** 

	All (n = 2100)	Men (n = 1036)	Women (n = 1064)	P for sex difference
	%	%	%	
How much in the last 12 months…				
Did this person make you feel good about yourself				**
Not at all/a little	14.0	11.6	16.4	
Quite a lot	40.6	38.8	42.3	
A great deal	45.4	49.6	41.3	
Did you share interests, hobbies				
Not at all/a little	19.9	20.2	19.6	*
Quite a lot	44.9	41.7	48.2	
A great deal	35.1	38.1	32.2	
Did you confide in this person				
Not at all/a little	29.9	26.7	33.0	*
Quite a lot	40.7	41.1	40.2	
A great deal	29.4	32.2	26.7	
Did talking to this person make things worse				
Not at all	79.7	80.6	78.8	
A little	18.1	17.6	18.7	
Quite a lot/a great deal	2.2	1.9	2.5	
Would you have liked to have confided more in this person				
Not at all	60.3	60.7	59.8	
A little	27.8	27.5	28.1	
Quite a lot/a great deal	11.9	11.7	12.1	
Did this person give you worries, problems and stress?				
Not at all	29.0	31.4	30.2	*
A little	57.2	50.2	53.6	
Quite a lot/a great deal	13.8	18.5	16.2	

**Table:** 

	Configural invariance	Metric (weak) invariance	Metric and scalar (strong) invariance
*Positive support (n = 3225)*			
Chi-square (degrees of freedom)	34.0 (21)	38.3 (25)	170.8 (29)
TLI	0.997	0.997	0.975
RMSEA (90% interval)	0.014 (0.004,0.022)	0.013 (0.002,0.021)	0.039 (0.033,0.045)
SRMR	0.014	0.016	0.026

*Negative support (n = 3218)*			
Chi-square (degrees of freedom)	217.1 (21)	219.4 (25)	315.83 (29)
TLI	0.894	0.912	0.888
RMSEA (90% interval)	0.054 (0.048, 0.060)	0.049 (0.043,0.055)	0.055 (0.050,0.061)
SRMR	0.040	0.041	0.046

TLI Tucker Lewis Index; RMSEA Root Mean Square Error of Approximation; SRMR Standardized Root Mean Square Residual.

**Table:** 

	PSQI score model 1^a^	PSQI score model 2^b^
Closest person identity	n = 2100	n = 2100
Spouse/partner	Reference	Reference
Son/daughter	0.39 (0.44)	0.31 (0.44)
Other relative	0.63 (0.65)	0.41 (0.65)
Friend/neighbour	0.93 (0.60)	0.67 (0.60)
Other	0.27 (0.66)	0.18 (0.65)
No-one	2.20 (1.02)⁎	2.06 (1.02)⁎
Female⁎		
Son/daughter	− 0.41 (0.47)	− 0.41 (0.46)
Other relative	− 0.35 (0.76)	− 0.33 (0.75)
Friend/neighbour	− 0.37 (0.66)	− 0.25 (0.66)
Other	1.84 (0.85)⁎	1.87 (0.85)⁎
No-one	− 0.50 (1.47)	− 0.30 (1.46)

a Model includes positive support, negative support, marital status, number people in household, educational attainment, longstanding limiting illness, leisure time physical activity, body mass index, caregiving.

b Additionally adjusted for affective disorder.

⁎ p < 0.05.

**Table:** 

	Positive support	Negative support
Fixed estimates	Mean (se)	Mean (se)
Intercept^a^ (estimate indicates support at age 68–69)	6.49 (0.05)	1.89 (0.04)
Age (estimate indicates overall change in support)	0.003 (0.004)	− 0.013 (0.003)⁎⁎
Female	− 0.248 (0.071)⁎	− 0.118 (0.059)
Female × change in support	− 0.012 (0.005)⁎	0.014 (0.005)⁎
Random estimates	Variance (se)	Variance (se)
Intercept	2.08 (0.10)	1.19 (0.08)
Age	0.0047 (0.0006)	0.0023 (0.0006)
Intercept-age covariance	− 0.026 (0.006)	− 0.026 (0.006)

a Centred at age 68.

⁎⁎ p < 0.001.

⁎ p < 0.05.
